# Clinical Clues and Diagnostic Workup of Cardiac Amyloidosis

**DOI:** 10.14797/mdcvj.1061

**Published:** 2022-03-14

**Authors:** Sajan S. Gill, Eric Fellin, Lisa Stampke, Yunazi Zhao, Ahmad Masri

**Affiliations:** 1 Knight Cardiovascular Institute, Oregon Health & Science University School of Medicine, Portland, Oregon, US; 2 Oregon Health & Science University School of Medicine, Portland, Oregon, US

**Keywords:** cardiac amyloidosis, systemic amyloidosis, immunoglobulin light chain, transthyretin, left ventricular hypertrophy, heart failure, aortic stenosis, bone scintigraphy, cardiac magnetic resonance imaging, endomyocardial biopsy

## Abstract

Cardiac amyloidosis is increasingly recognized as an underlying cause of left ventricular wall thickening, heart failure, and arrhythmia with variable clinical presentation. Due to the subtle cardiac findings in early transthyretin cardiac amyloidosis and the availability of therapies that can modify but not reverse the disease progression, early recognition is vital. In light chain amyloidosis, timely diagnosis and treatment can significantly improve survival. In this manuscript, we review the clinical, imaging, and electrocardiographic clues that should raise suspicion for cardiac amyloidosis and provide a simplified diagnostic workup algorithm that ensures an accurate diagnosis. The evolution of the noninvasive diagnosis of cardiac amyloidosis has significantly influenced our understanding of disease prevalence, presentations, and outcomes. However, clinical recognition of clues and red flags remains the most important factor in advancing the care of patients with cardiac amyloidosis.

## Introduction

**Figure 1:**
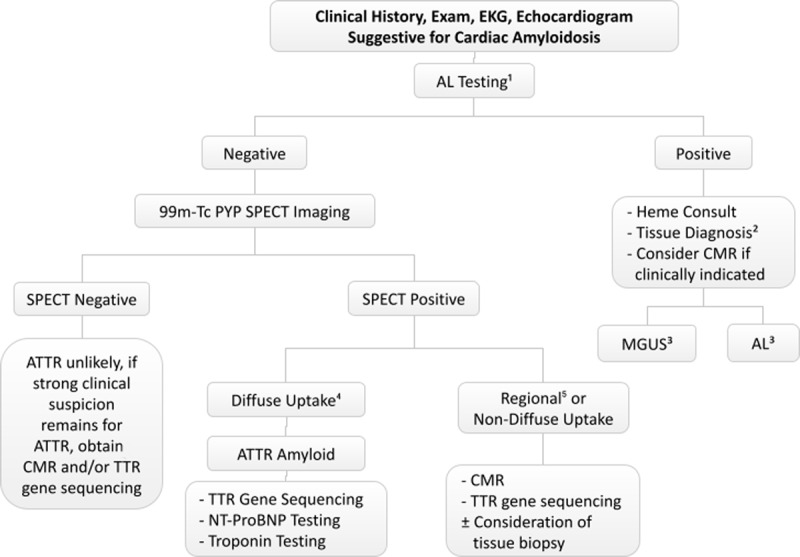
Simplified diagnostic algorithm for suspected cardiac amyloidosis. EKG: electrocardiogram; AL: immunoglobulin light chain; Heme: hematology; CMR: cardiac magnetic resonance imaging; Tc-99m PYP: technetium-99m pyrophosphate; SPECT: single-photon emission computed tomography; MGUS: monoclonal gammopathy of undetermined significance; ATTR/TTR: transthyretin; NT-proBNP: N-terminal prohormone of brain natriuretic peptide

Systemic amyloidosis is caused by the extracellular deposition of misfolded protein in multiple organs and systems. The two most prevalent amyloid types with common cardiac involvement are immunoglobulin light chain and transthyretin (ATTR) amyloidosis. These can overlap in presentation but differ in diagnostic pathway and therapeutic approaches^1^; hence, it is crucial to identify the underlying subtype of cardiac amyloidosis. In this review, we discuss the clinical clues that should raise suspicion for cardiac amyloidosis (CA) in patients presenting in outpatient settings, and we provide a patient-centered diagnostic workup that ensures an accurate diagnosis (***Figure 1***).

Light chain amyloidosis (AL-CA) is caused by sporadic deposition of kappa or lambda light chains in tissue and therefore does not follow any known inheritance pattern. The disease is one of the plasma cell dyscrasias and found in 10% of patients with multiple myeloma.^2^ AL-CA is relatively uncommon, with an incidence of 3 to 9 per million person-years.^3^ Across all forms of amyloidosis, misfolded proteins are often more prevalent in older individuals, possibly due to the changes in physiological microenvironments seen with aging.^1^ The age at diagnosis varies, although the mean age of AL-CA diagnosis is approximately 57 years old.^4,5^ AL-CA is historically more readily recognized compared to ATTR, often with a wide-ranging presentation depending on which organ is involved. Symptoms may include heart failure, weight loss, nephrotic syndrome, macroglossia, and GI disturbances, among others.^6^

Transthyretin is a thyroxine and retinol transport protein predominantly produced in the liver, choroid plexus, and retina and is found circulating in plasma and cervical spinal fluid.^7^ ATTR amyloidosis can result from an amino acid change, leading to variants (ATTRv) that precipitate transthyretin tetramer instability, or can occur despite having normal protein structure, as in wild type ATTR (ATTRwt).^8^ These two types are closely related in pathophysiology, both showing deposition of fibrils in different organs and systems, but the distinction comes in their presentation and currently available therapies.

As the name suggests, ATTRwt is a sporadic disease of older individuals, most often seen in White males, with prevalence increasing significantly (12-25%) in people over 80 years.^9^ ATTRwt predominantly presents as heart failure with features of restrictive and infiltrative cardiomyopathy, with additional history clues including orthopedic manifestations such as prior carpal tunnel release, spinal stenosis, and neuropathy.^10^ ATTRv is inherited through an autosomal dominant pattern with over 100 different associated variants.^11^ The severity, age of onset, and symptomology of the disease is dependent on the particular variant and geographical location.^12^ In ATTRv, early onset is considered before the age of 50, and incidence varies geographically in endemic versus nonendemic areas.^13,14^ In the United States, there is a higher prevalence of the p.Val142Ile mutation among people of African and Afro-Caribbean ancestry but with variable penetrance.^15^ This mutation is of particular interest given its prevalence and predominantly cardiac phenotype.^16,17^ Overall, ATTRv has a rather heterogeneous presentation depending on the underlying variant and can emerge with a polyneuropathy phenotype, cardiomyopathy phenotype, or mixed.^6,18^

Due to the varying and at times nonspecific symptomatology in patients with amyloidosis, it is important to pay attention to minor clues in a patient’s history, such as previous orthopedic or neurological conditions that were present years prior to presentation, and carefully evaluate left ventricular hypertrophy (LVH) even in the presence of hypertension. ***Table 1*** summarizes extracardiac symptoms and disease manifestations that should alert the clinician to consider amyloidosis in the differential.^19,20,21^

**Table 1:** Extracardiac findings in amyloidosis that should prompt workup in patients presenting with heart failure.^19,20,21^


PRESENTATION	AL AMYLOID	ATTR AMYLOID

Foamy urine	✔	**—**

Hepatosplenomegaly	✔	**—**

Macroglossia	✔	**—**

Purpura (periorbital, neckline)	✔	**—**

Arthropathy	✔	**—**

Skin bruising	✔	**—**

Autonomic dysfunction (intestinal motility/orthostatic hypotension)	✔	✔*

Dysesthesia	✔	**—**

Carpal tunnel syndrome (often bilateral)	**—**	✔

Biceps tendon rupture	**—**	✔

Lumbar spinal stenosis	**—**	✔

Trigger finger	**—**	✔

Vitreous deposits	**—**	✔

Constipation/diarrhea	✔	✔

Unexplained weight loss (dysphagia, malabsorption)	✔	✔

Polyneuropathy	✔	✔*


* More common in this subtype

## Cardiac Phenotype (Heart Failure)

In the cardiac phenotype, amyloidosis can cause a wide range of presentations; however, the classic presentation is often cardiomyopathy with restrictive hemodynamics and heart failure.^22^ The cardiac phenotype typically starts with subclinical extracellular deposition of amyloid fibrils, with progressive increase in ventricular wall thickness, atrial dilatation, arrhythmias, and conduction system abnormalities.^20,23^ The cardiac phenotype is the leading cause of mortality, with varying survival rates depending on the stage of cardiac involvement.^24^ For example, in untreated advanced AL-CA, the mean survival is less than 6 months after development of heart failure.^25^ Early recognition of cardiac involvement in amyloidosis is vital given the high morbidity and mortality coupled with therapeutics that frequently halt the progression of disease but do not reverse it.^22^

Perhaps the most important aspect is developing a systematic approach to evaluating systemic signs, symptoms, and diagnostic findings that could provide clues to the diagnosis, which, in the era of electronic medical record systems, can be integrated as a systematic checklist for every new patient with heart failure. ***Table 2*** provides some clues from cardiac involvement in amyloidosis.^17,19,20,26,27,28,29,30,31,32^

**Table 2:** Clinical, echocardiographic, and EKG clues to cardiac amyloidosis.^17,19,20,26,27,28,29,30,31,32^ AL: amyloid light chain; BB: beta blocker; ACEi: angiotensin converting enzyme inhibitor; ARB: angiotensin receptor blocker; ARNI: angiotensin receptor neprilysin inhibitor; LVEF: left ventricular ejection fraction; GLS: global longitudinal strain; EKG: electrocardiogram; LV: left ventricle; MI: myocardial infarction


CLINICAL FINDINGS

Proteinuria (AL)

Hepatosplenomegaly (AL)

Syncope

Unexplained weight loss, fatigue, cachexia

Orthostatic hypotension

Progressive decline of blood pressure, or the need for less anti-hypertensive medications over time

Inability to tolerate standard heart failure therapies (BB, ACEi/ARB, ARNI) or rate control strategy in atrial fibrillation

**ECHOCARDIOGRAPHIC FINDINGS**

Left ventricular hypertrophy particularly when associated with relative apical sparing pattern on global longitudinal strain analysis

Restrictive diastolic filling pattern

Left ventricular ejection fraction to global longitudinal strain ratio (LVEF/GLS) > 4.1

Aortic stenosis

Mitral annular tissue Doppler S’ < 6 cm/s

Left ventricular ejection fraction 50% ± 5%

Low QRS voltage to LV mass ratio

Thickening of aortic and mitral valves and intra-atrial septum

Pericardial effusions

Average apical/basal longitudinal strain ratio > 2

Atrial enlargement

Normal/small LV cavity size

**EKG FINDINGS**

Low voltage (QRS < 1 mV in precordial and < 0.5 mV in extremity leads)

Pseudoinfarct patterns without known prior MI (QS waves in any two consecutive leads)


While the sensitivity and specificity of electrocardiograms for cardiac amyloidosis diagnosis is low, the combination of red flags from the patient’s history and electrocardiogram, in the setting of LV hypertrophy, should prompt an evaluation for cardiac amyloidosis.^33,34^ A history of hypertension should not discourage such an evaluation since hypertension is common in patients with a history of ATTR.^35^ Once clinical suspicion is raised, pretest probability should drive targeted evaluation.

## Aortic Stenosis

Coexistent aortic stenosis (AS) and ATTR cardiomyopathy (ATTR-CM) is a relatively recent finding, and there are no mechanistic studies to directly explain the high prevalence of ATTR-CM in AS.^30,36^ The coexistence of these conditions represents a unique challenge since AS can also lead to increased LV wall thickness and abnormal strain pattern. However, this also represents a unique opportunity to apply screening for red flags in patients presenting with AS, akin to those presenting with heart failure, to identify those with underlying ATTR-CM (***Table 2***). This is especially important given the comparable mortality between AS patients who underwent transcatheter aortic valve replacement (TAVR) and patients with AS and ATTR-CM who underwent TAVR without other treatments for ATTR-CM.^37^ Nitsche et al. recently reported on a clinical risk score that can be used to identify ATTR-CM by narrowing down red flags, which include carpal tunnel syndrome, right bundle branch block, ≥ 85 years of age, elevated high sensitivity troponin T ≥ 20 ng/L, having an interventricular septum thickness ≥ 18 mm, E/A ratio ≥ 1.4, and, when feasible to perform, a Sokolow index < 1.9 millivolt.^38^ Ultimately, the message is to recognize that ATTR-CM coexists with AS, and an evaluation for ATTR-CM red flags in all patients with AS is necessary to improve the care of such patients, especially given the availability of therapeutics.

## Laboratory Evaluation

Currently, no single laboratory test can definitively rule in or out cardiac amyloidosis. When used within the clinical context, elevated N-terminal prohormone of brain natriuretic peptide (NT-proBNP) in patients with stable symptoms in the nonacute setting can be a red flag when it is discrepant with New York Heart Association (NYHA) class. Similarly, chronically elevated troponin is a marker of subclinical myocardial injury and, in the absence of other explanation, should prompt workup for amyloidosis in the presence of LVH.^39,40^ These markers, in addition to glomerular filtration rate, are the backbone of prognostic models in both AL-CA and ATTR-CM.^41,42,43,44^

Additionally, with the overlap between AL and ATTR presentation, it is important to rule out AL-CA via laboratory studies early in the evaluation, although at times, for practicality purposes, ruling out AL and obtaining technetium-99m pyrophosphate (Tc-99m PYP) scintigraphy occur simultaneously. All patients with CA phenotype should undergo direct serum and urine immunofixation electrophoresis and light chain quantification to screen for AL-CA with a > 95% sensitivity.^45,46^ Local teams should work with their respective labs to ensure that direct immunofixation is performed on all samples, irrespective of the results of protein electrophoresis, rather than only as a reflex immunofixation to samples with abnormal protein electrophoresis. It should be noted that patients with ATTR-CM have a high prevalence of monoclonal gammopathy of undetermined significance (MGUS, defined by any abnormalities in the previously mentioned laboratory work), and this should prompt careful evaluation in conjunction with a hematologist to rule out AL-CA and risk stratify MGUS.^45,47,48^ Light chains are renally cleared, and in the setting of chronic kidney disease, both kappa and lambda light chain levels are elevated (kappa > lambda), which led to the introduction of a “renal” free light chain ratio to account for alterations in renal clearance in these patients.^49,50,51^ Given the complexities of patients presenting with CA, a granular evaluation of these patients is still indicated.

In ATTR-CM, transthyretin (formerly known as prealbumin) levels can be useful for risk stratification and show early promise for assessing treatment response in patients on tafamidis.^52,53^ Additionally, retinol binding protein 4, which binds to the transthyretin tetramer, has shown diagnostic potential when combined with additional clinical factors in identifying p.Val142Ile ATTRv-CM.^54^

## Diagnostic Imaging

The two most common imaging modalities used after echocardiography in the workup of LVH are cardiac magnetic resonance imaging (CMR) and bone scintigraphy (specifically Tc-99m PYP, 99mTc-3,3-diphosphono-1,2-propanodicarboxylic acid, and Tc-99m hydroxy-methyl-diphosphonate). CMR can be viewed as part of the routine workup of patients with LVH or can be specifically used in the workup of patients with suspected CA. Pretest probability should guide which imaging test to pursue after echocardiography. In patients who have undifferentiated LVH or appear to be at low to intermediate suspicion of CA, CMR is the appropriate next step in evaluating LVH. In patients who have clear high pretest probability for ATTR-CM based on clinical and echocardiographic evaluation, bone scintigraphy is used to confirm ATTR-CM after ruling out AL amyloid cardiomyopathy.^55^

### Cardiac Magnetic Resonance Imaging

CMR provides a wealth of cardiac data including anatomy, function, and tissue characterization, with the constellation of findings being highly sensitive and specific for detecting CA.^56^ Aside from the typical features that can be seen, two main CMR sequences provide diagnostic and mechanistic understanding into the underlying burden of disease.

#### Late gadolinium enhancement imaging (LGE)

**Figure 2:**
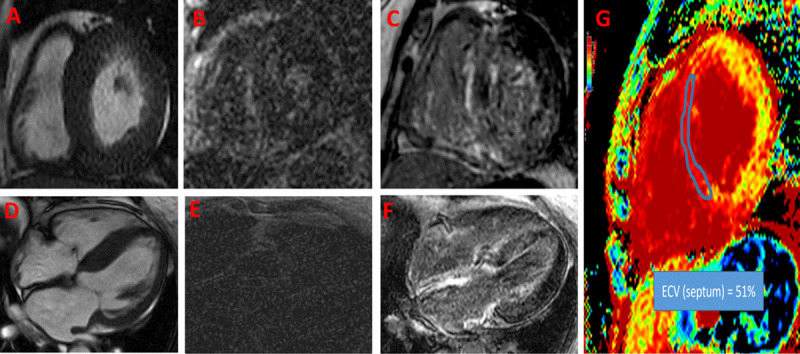
Cardiac magnetic resonance imaging (CMR) of a patient presenting with dyspnea and found to have left ventricular hypertrophy. First set of images (**A, B** and **E**) were obtained prior to presenting to our practice. Image **A** shows concentric left ventricular hypertrophy. Due to abnormal gadolinium kinetics and selecting an inappropriately low inversion time, late gadolinium enhancement (**B**) short axis and (**E**) 4-chamber views were not interpretable. (**D**) Repeat CMR shows severe asymmetrical septal hypertrophy on 4-chamber view; with choosing an appropriate inversion time for late gadolinium enhancement imaging, there was global enhancement of the left ventricle (sparing anterior and anterolateral segments), right ventricle, and both atria (**C, F**). Image **G** shows significant expansion of the extracellular volume (ECV) fraction (51% in the septum), which can be reliably obtained even if late gadolinium imaging sequences are suboptimal.

In amyloidosis, LGE is reflective of amyloid fibrils occupying the extracellular space and the associated subendocardial fibrosis.^57^ The presence and pattern of LGE provides important diagnostic clues to the extent of CA. The main LGE patterns are diffuse subendocardial LGE and transmural LGE. A common issue when performing LGE imaging is abnormal gadolinium kinetics, where the myocardium and blood T1 values are similar because of high myocardial uptake and fast blood pool washout, which makes it harder to null the myocardium for acquisition of phase-sensitive inversion recovery sequences.^58^ Although abnormal gadolinium kinetics can provide diagnostic clues, it can cause confusion in interpreting the images due to poor LGE technique (***Figure 2A-F***). Routine use of native T1 mapping and extracellular volume (ECV) quantification can rescue these shortcomings (***Figure 2G***).

#### T1 mapping and ECV

**Figure 3:**
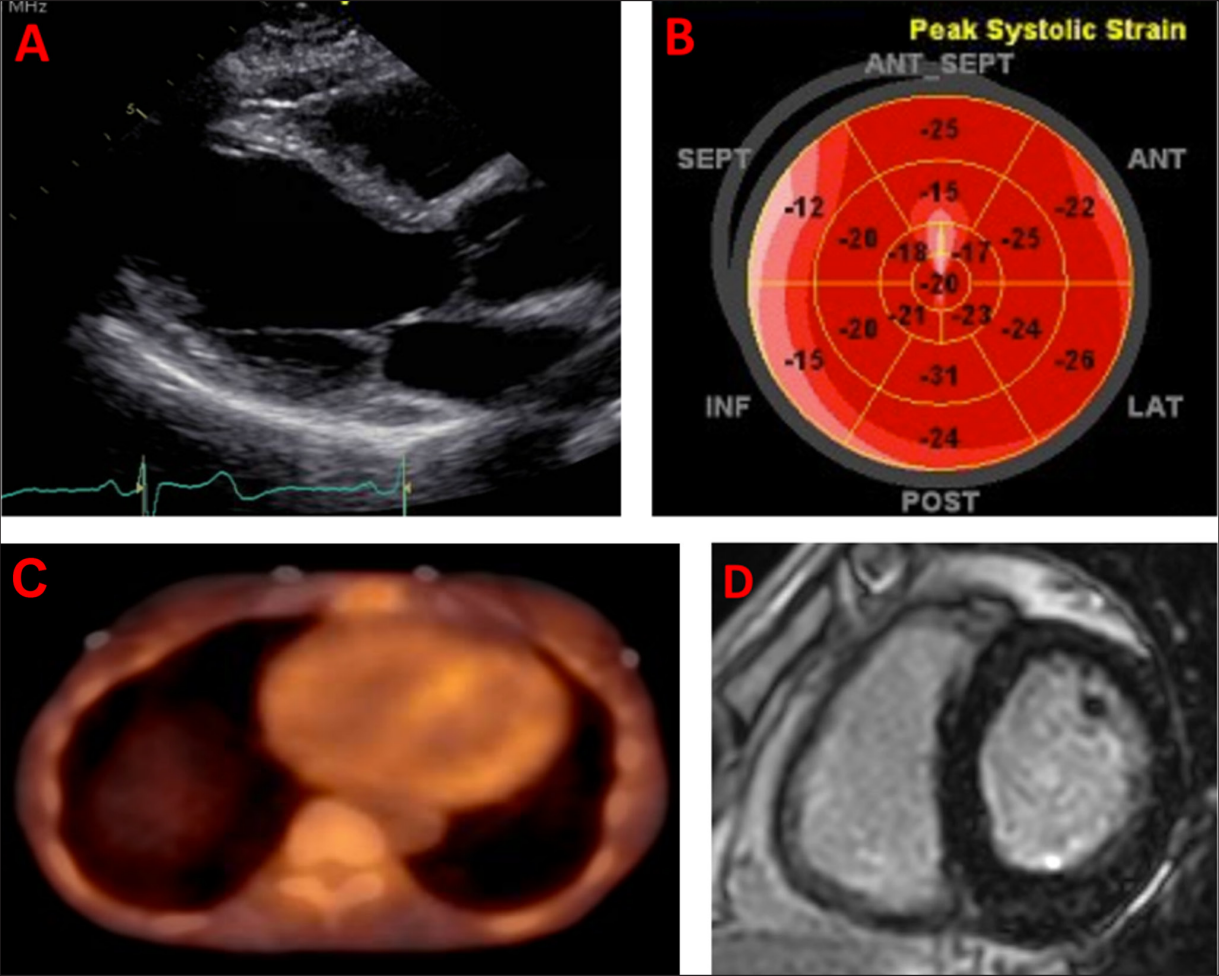
Multimodality imaging in the workup of a 70-year-old patient who is a carrier for the p.V50M variant and who presented with exertional shortness of breath and palpitations. (**A**) Echocardiogram parasternal long-axis window showing normal left ventricular wall thickness. (**B**) Depressed longitudinal strain, particularly in the basal septal segments (absence of apical sparing pattern). (**C**) 99m-technetium pyrophosphate single photo emission computed tomography showing diffuse uptake of the tracer in the myocardium. (**D**) Cardiac magnetic resonance imaging with phase-sensitive inversion recovery sequences obtained 15 minutes post gadolinium show minimal late gadolinium enhancement, but dedicated sequences showed elevated native T1 (1130 millisecond, 1.5 Tesla) and extracellular volume fraction (39%).

Progressive amyloid fiber deposition leads to progressive expansion of the extracellular space, which can be estimated using CMR. Native T1 mapping can offer clues regarding the presence of CA without using gadolinium.^59^ ECV, which requires both native and post-gadolinium T1 map, can also be used as a surrogate for total amyloid mass in the myocardium.^60^ Rarely, certain early amyloidosis phenotypes, especially in patients with hereditary ATTR, can present without significant LVH and with minimal or absent LGE but with elevated T1 mapping and ECV (***Figure 3***). Hence, one should always attempt to acquire these sequences in patients referred with LVH or suspicion for CA.

It is worth noting that CMR cannot differentiate between subtypes of CA. Based on the recent multisocietal expert consensus recommendations for multimodality imaging in CA, typical imaging findings of CA on CMR should be accompanied by either histological confirmation (cardiac or extra-cardiac) or by bone scintigraphy and laboratory studies for AL-CA.^61^

### Bone Scintigraphy

The resurrection of Tc-99m PYP scintigraphy and a subsequent multicenter study in 2016 have revolutionized the approach to diagnosing ATTR-CM.^62^ While planar imaging has been historically used, recent advances have shown how myocardial single-photon emission computed tomography (SPECT) imaging is essential to define myocardial involvement and exclude false positive scans due to blood pooling of the tracer.^63,64,65^ In addition, SPECT/computed tomography in particular provides excellent anatomical landmarks that help differentiate blood pooling versus myocardial uptake in milder and regional forms of tracer uptake.^66^ Despite its limitations, in a population with a high clinical pre-test probability for ATTR-CM, grades 2 to 3 on planar imaging combined with a negative serum evaluation for AL-CA yield a 100% positive predictive value.^55^ The use of Tc-99m PYP with SPECT can be a successful strategy in making a nontissue diagnosis of ATTR-CM when clear diffuse uptake of the tracer is present. In all other atypical imaging findings, supportive evidence should be sought from other imaging tests or tissue biopsy to avoid misdiagnosis of ATTR-CM.

## Tissue and Endomyocardial Biopsy

Historically, endomyocardial biopsy was required to diagnose and subtype cardiac amyloidosis. With advances in diagnostic imaging and evaluation of CA, endomyocardial biopsy is not required routinely for ATTR-CM patients. Biopsy in ATTR-CM is indicated in the following cases: (1) when one cannot reliably differentiate between AL and ATTR-CM, such as abnormal monoclonal protein workup in elderly individuals who are at risk for ATTR-CM or in conjunction with abnormal bone scintigraphy; (2) when imaging has atypical findings, such as borderline or negative nuclear scintigraphy in suspected patients with ATTR-CM; or (3) to evaluate for other less common forms of amyloidosis. In AL-CA, endomyocardial biopsy is more frequently required in the absence of histological confirmation from an extra-cardiac site. The demonstration of Congo red stained extracellular amorphous material in the presence of an amyloid clinical phenotype is diagnostic of the disease, with mass spectroscopy providing unique capabilities of subtyping the amyloid protein.^67^

## Transthyretin Gene Sequencing

In patients deemed to have ATTR-CM, transthyretin gene sequencing is essential to differentiate ATTRv-CM from ATTRwt-CM. It is vital to differentiate between the two subtypes because there are implications with regard to understanding disease severity, family screening, and utilization of approved therapies.^22,68,69^ If a patient is genotype positive for a pathogenic variant, it is vital to provide genetic counseling and assess family members either for the phenotype or via cascade genetic screening. ATTRv has an autosomal dominant inheritance pattern that is age dependent with variable penetrance.^11^ Each pathogenic variant has its own unique biochemical effects and phenotype. Generally, it is recommended to start genetic testing at least 10 years prior to the onset of clinical disease in a family member or earlier if signs of clinical disease appear.^1^

## Conclusion

Cardiac amyloidosis, particularly ATTR-CM, is common in elderly patients with heart failure. Red flags exist and can be systematically incorporated into one’s own practice as simple screening tools to improve the pretest probability. It is essential to differentiate ATTR-CM from AL-CA given the vastly different treatment approach. As the noninvasive diagnosis of CA continues to evolve, improvement in the diagnosis rate of ATTR-CM with less reliance on endomyocardial biopsy as well as availability of novel therapeutics promise a better future for patients living with ATTR-CM.

## Key Points

- Transthyretin cardiac amyloidosis (CA) is a common cause of heart failure in elderly patients. Enhancing pretest probability by clinical screening of red flags improves diagnostic yield of testing.
- Transthyretin amyloid cardiomyopathy coexists with aortic stenosis.
- Light chain amyloidosis should be ruled out in all patients with CA phenotype using serum free light chain assay and direct serum and urine immunofixation electrophoresis.
- A modern approach to the noninvasive diagnosis of transthyretin CA using bone scintigraphy requires imaging with single-photon emission computed tomography.
- Cardiac magnetic resonance imaging has diverse roles in the imaging cascade of patients with left ventricular hypertrophy including CA.

## CME Credit Opportunity

Houston Methodist is accredited by the Accreditation Council for Continuing Medical Education (ACCME) to provide continuing medical education for physicians.

Houston Methodist designates this enduring material for a maximum of .25 AMA PRA Category 1 Credit™. Physicians should claim only the credit commensurate with the extent of their participation in the activity.

Click to earn CME credit: https://cvent.me/0KEEPx.
